# Detecting patterns of accessory genome coevolution in *Staphylococcus aureus* using data from thousands of genomes

**DOI:** 10.1186/s12859-023-05363-4

**Published:** 2023-06-09

**Authors:** Rohan S Mehta, Robert A Petit, Timothy D Read, Daniel B Weissman

**Affiliations:** 1 Department of Physics, Emory University, Atlanta, GA USA; 2 Division of Infectious Diseases, Department of Medicine, School of Medicine, Emory University, Atlanta, GA USA; 3 Wyoming Public Health Laboratory, Cheyenne, WY USA; 4 Department of Human Genetics, School of Medicine, Emory University, Atlanta, GA USA

**Keywords:** Genomics, Genetic interaction, Horizontal gene transfer, *Staphylococcus aureus*, Software, Microbial genomics

## Abstract

Bacterial genomes exhibit widespread horizontal gene transfer, resulting in highly variable genome content that complicates the inference of genetic interactions. In this study, we develop a method for detecting coevolving genes from large datasets of bacterial genomes based on pairwise comparisons of closely related individuals, analogous to a pedigree study in eukaryotic populations. We apply our method to pairs of genes from the *Staphylococcus aureus* accessory genome of over 75,000 annotated gene families using a database of over 40,000 whole genomes. We find many pairs of genes that appear to be gained or lost in a coordinated manner, as well as pairs where the gain of one gene is associated with the loss of the other. These pairs form networks of rapidly coevolving genes, primarily consisting of genes involved in virulence, mechanisms of horizontal gene transfer, and antibiotic resistance, particularly the SCC*mec* complex. While we focus on gene gain and loss, our method can also detect genes that tend to acquire substitutions in tandem, or genotype-phenotype or phenotype-phenotype coevolution. Finally, we present the R package DeCoTUR that allows for the computation of our method.

**Supplementary Information:**

The online version contains supplementary material available at 10.1186/s12859-023-05363-4.

## Introduction

Interactions between genes are a major part of evolution, but they are fundamentally difficult to study due to the combinatorial explosion of the number of possible interactions [1, 2]. In bacteria, widespread horizontal gene transfer creates a much wider range of potential genetic backgrounds and genetic interactions [3]. Detecting gene-gene interactions without performing large numbers of assays requires the development of computational techniques that can handle the necessary volume of genomic data to find signatures in natural genetic diversity.

Methods for finding interactions at the level of genes generally perform Genome-Wide Association Studies (or GWAS) to detect relationships between genes and phenotypes. This approach has been widely used in human populations, and while there have been successes (the first of which was [4]; see [5]), GWAS inference in humans is often complicated by the existence of population structure—systematic differences in allele frequencies among subgroups in a population [e.g. 6, 7]. This is even more of a problem in bacterial populations, which often have stronger population structure due to their limited and biased recombination [8–10]. Thus, it is usually insufficient to simply transfer existing association detection methods from humans to bacteria, and new methods must be developed that take into account the structure of bacterial populations.

There are several existing approaches to detect genotype-phenotype associations in bacteria, the earliest of which are reviewed in [8]. The software PLINK [11], which is frequently used in human GWAS studies, has also been applied to bacterial datasets [12–14]. Approaches developed specifically for bacteria include those based on regression [15–18] and those based on phylogenetic convergence [9]. Techniques that explicitly take phylogenetic information into account fare better in highly clonal bacterial systems [16, 18].

Methods that use phylogenetic convergence are based on homoplasic events on a phylogeny. The package hogwash [19] implements two methods based on ancestral state reconstruction: phyC (introduced by [20]) and a more stringent method that was introduced by [21]. The package treeWAS [22] pairs ancestral state reconstruction with simulation given a homoplasy distribution to compute three different tests of association: one that only uses leaf data and is equivalent to the method proposed by [23], one that is equivalent to phyC [20], and one that is novel and takes into account co-occurance times along the tree. Finally, Scoary [24], uses the method of pairwise comparisons [25] to find the minimum number of necessary independent co-emergences of two genes given a phylogeny and evaluates association based on this number. These methods are generally computationally demanding, and indeed were left out of a recent simulation study comparing various bacterial GWAS techniques precisely for this reason [18].

Computational complexity is a problem for all GWAS-style methods when applied to gene-gene interactions, which are built for many-to-one association tests and not many-to-many tests. While in principle all current published GWAS-style methods could be used to broadly detect gene-gene interactions (by treating the presence or absence of a gene as a “phenotype”), they are in general not built for comparing multiple sets of genes against each other simultaneously and running them for pairwise comparisons of large numbers of genes becomes prohibitively slow. Thus, it is useful to specifically design methods for detecting interactions between genes via co-occurrence. Pantagruel [26] estimates gene trees and evaluates the co-incidence of events on gene trees under a species tree. CoPAP [27, 28] simulates gain and loss events for pairs of genes along a phylogeny under various coevolutionary models. [29] use a maximum likelihood method developed by [30] to identify genes that have related gain and loss patterns. Most of these approaches use specified evolutionary models, which can become unwieldy over large datasets as tree size grows. The recent method Coinfinder [31] avoids using a full phylogenetic simulation or likelihood analysis by computing the phylogenetic statistic of lineage independence D [32] along with a simple statistic of co-incidence to determine putative gene-gene interactions. Finally, methods originally designed to detect epistasis—such as superDAC [33] and SpydrPick [34]—can be adapted to apply to gene gain and loss as well. As we demonstrate in this study, these relatively model-free methods have different strengths and weaknesses in different settings, and it is useful to have a method that has a tunable parameter that can be adjusted to work well across multiple different settings.

Here, we introduce a new method for finding associations between genes in bacterial populations, specifically tailored to accommodate datasets with greater than 1,000 samples, and with a parameter that can be tuned to accommodate many differently sized datasets, by sidestepping a full phylogenetic analysis entirely. This method, which we call DeCoTUR (Detecting Coevolving Traits Using Relatives), is based on the idea that a strong signal of biological association can be inferred if closely related individuals differ in their gene presence-absence states in the same way. In our approach, we first identify pairs of closely related individuals. The number of these close pairs is the tunable parameter. We then find pairs of genes for which, when one gene is gained or lost between a pair of closely related individuals, the other gene is frequently gained or lost as well. We apply our method to the Staphopia database [35]—which contains over 40,000 publicly available *Staphylococcus aureus* genomes—to detect correlated gain and loss between pairs of accessory genes. The number of such coincident gain/loss events determines a gene pair’s “coevolution score”. We test for interactions by comparing this coevolution score to what would be expected if the two genes were gained and lost independently. With this method, we find interactions between genes involved in a wide variety of functions, including antibiotic resistance, virulence, pathogenicity, phage interactions, mobile genetic elements, and others. The majority of these interactions are positive associations, i.e., pairs of genes that are gained and lost together, rather than substituting for each other. We find many interactions between closely linked genes that are likely co-transferred, particularly among genes related to antibiotic resistance. We also find interactions between genes that are not closely linked, especially among genes related to virulence. The coevolution of these pairs is likely to involve multiple transfer events and be driven by epistasis (functional interactions between genes) or correlated selection across environments. Finally, we introduce the R package DeCoTUR (https://github.com/weissmanlab/decotur) that allows the computation of our coevolution score.

## Methods

### Data

We downloaded all public samples from the Staphopia database [35], for a total of 42,949 samples. We used the core genome of shared genes determined by [35] to compute nucleotide divergences between the samples and we removed 10,308 samples that were identical in core genome sequence and accessory genome composition to at least one other sample. We used each sample’s multi-locus sequence type (MLST, provided by Staphopia) and the publicly-available pubMLST database (https://pubmlst.org/saureus/) to determine its clonal complex (CC). For a breakdown of sample size by clonal complex, see Additional file 1: Fig. S1 and Section A. We also computed coevolution scores among antibiotic resistance phenotypes across the whole database obtained from ARIBA predictions [36] in Staphopia.

### Finding close pairs of individuals

We determined closely-related (i.e. “close”) pairs of samples based on the distribution of distances in a pairwise distance matrix—computed using Hamming distances on the concatenated core genome—of all considered samples. This procedure requires a choice of distance cutoff, with pairs of samples whose pairwise distance is below this cutoff are considered to be “close”. In principle, this cutoff can be tuned to whatever scale is of interest, or to match the number of close pairs to the available computational power. Note that as sample sizes increase, the computational expenditure of this procedure can be mitigated in two ways: first, pairwise distances need only be calculated between individuals that could potentially be closely related to each other, and second, a less direct approach such as using Mash distances [37] can be used.

We computed pairwise distances between all samples within each clonal complex (Additional file 1: Fig. S2, Section B). To choose a distance cutoff which would not dramatically overrepresent some clonal complexes and underrepresent others, we chose a distance cutoff of 5 × 10^−4^ (Additional file 1: Fig. S3, Section C). For the three clonal complexes with > 1,000,000 close pairs below this cutoff (5, 8, and 22) we downsampled close pairs to match the average representation of the other clonal complexes given this distance cutoff. We then randomly sampled 10,000 of the resulting close pairs to get to our final set of close pairs. The analysis in Section E demonstrates that the specific choice of cutoff does not dramatically affect the overall results.

### Gene annotation, clustering, and filtering

To annotate the genomes, we processed contig sequence data from Staphopia with prokka [38]. We then used Panaroo [39] on a subsample of size 10,000 of these processed samples for pangenome clustering, with the gene presence-absence output from Panaroo as our gene presence-absence matrix. Panaroo clusters genes into families while splitting up paralogs by default. We disabled the option to find genes missed by prokka for time purposes. We combined gene families that had the same names up to a permutation in order. For each analysis, we only include genes that have at least two of the less frequent state (presence or absence) in the set of samples used in close pairs. These are the only genes with sufficient presence-absence polymorphism to potentially show a signal of coevolution.

### Computing the coevolution score

**Fig. 1:**
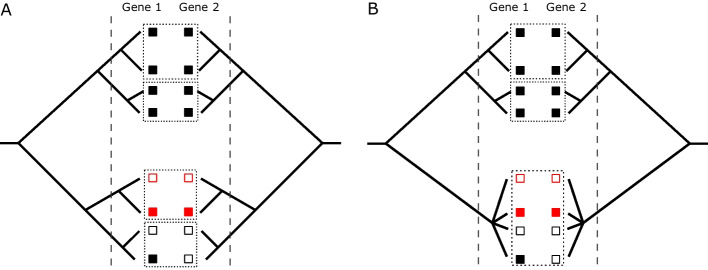
Two examples of the coevolution score computation for a pair of genes (left and right trees in each panel).** A** an example with all disjoint close pairs.** B** An example with an unresolved polytomy “bush,” in which all individuals present are close pairs with each other. The vertical dashed lines indicate the distance cutoffs used to determine close pairs. Filled squares indicate presence of a gene, empty squares indicate its absence. Dashed boxes indicate individuals that are in close pairs with each other. In both** A** and** B**, there is exactly one close pair of individuals (in red) that is polymorphic for both genes, indicating recent gain/loss, so only that close pair contributes to the score. The genes differ in the same way (the top red individual has neither gene, the bottom red individual has both), so this contributes to the *positive* score for the gene pair. In** A**, the single close pair contributes a value of 1 to the positive score. In** B**, this close pair is part of a bush of ${4 \atopwithdelims ()2} = 6$ close pairs, so it contributes only $\frac{1}{6}$ to the positive score. The more ancient event that produced the difference between the top clade (where both genes are present in all individuals) and the bottom clade (where both genes are mostly absent) does not contribute to the score. Note that our method does not actually use the trees, only which pairs of individuals are closely related

Here we will outline how we test for coevolution between a specific pair of genes, gene 1 and gene 2. To compute the coevolution score, we test each pair of closely related individuals *i* and *j* for evidence of coevolution. Most pairs of close relatives will necessarily be uninformative: for each gene, they will either both have the gene or both lack it, simply by virtue of being closely related. But for genes that are frequently gained and lost, there will be some pairs of close relatives that differ in the focal genes, and these are the pairs that can contribute to the score. Let $P_{n,k}$ be an indicator variable for the presence of gene *n* in individual *k*, e.g., $P_{1,i} = 1$ if individual *i* has gene 1 and 0 otherwise. If one individual has both genes and the other individual has neither, i.e., $(P_{1,i}, P_{1, j}, P_{2, i}, P_{2,j}) = (1, 0, 1, 0)$ or (0, 1, 0, 1), then we add + 1 to the score representing a positive association between the genes. Conversely, if one individual has only one gene and the other individual has only the other, i.e., $(P_{1, i}, P_{1, j}, P_{2, i}, P_{2,j}) = (1, 0, 0, 1)$ or (0, 1, 1, 0), then we add + 1 to the score representing a negative association between the genes. We compute two separate scores, one for each of these two types of associations. Figure 1 provides an example situation which illustrates how the score focuses on recent co-incident evolutionary events (represented by the red samples in Fig. 1), while omitting older evolutionary events. Additional file 1: Fig. S4 (Section D) displays the distribution of discordant close pairs for each gene. These discordances are approximately exponentially distributed with a mean of 108 discordances per 10,000 close pairs. Thus, the overall rate of gene gain/loss at the scale of these close pairs is about 1% per pair.

The phylogenies of clonal complexes in *S. aureus* often feature multiple clusters of extremely closely related individuals that form “bushes”—or in extreme cases, polytomies—in which it is difficult to tell which samples are most closely related (see Additional file 1: Fig. S6, Section F), and for which the specific tree structure may be difficult to infer accurately. Rather than trying to resolve these bushes, we adjust the value of the contribution for each close pair based on the size of the bush it comes from. Specifically, we partition all the samples into groups where two samples are in the same group if they form a close pair. If pair *k* is in a group with $n_k$ total pairs, then we divide the contribution of that pair to the score by $n_k$. In other words, the maximum total contribution of each bush to the score is 1. This is a highly conservative estimate of the amount of coevolution in bushes; it treats a bush as if it were an unresolved polytomy and ignores any tree structure inside the bush that may otherwise indicate a coevolutionary signal. In Fig. 1B, there are three bushes, two of size 2 and one of size 4. Only the size 4 bush contributes to the score, and the contribution to the score of that bush is 1/6, as one of the six close pairs in that bush (the red pair) contains a pattern that contributes to the score. Contrast this to the situation in Fig. 1A, in which the only bush that contributes to the score is of size 2, so its contribution is 1.

Because our method is based on genetic diversity, it necessarily has the most power to detect coevolution among genes that are at intermediate frequencies. But because we focus on recent/ongoing evolution, the power to detect coevolution does not just depend only on the frequency of a gene in the sample, but also on its distribution. For genes that are essentially exclusively clonally inherited and whose polymorphism corresponds to a deep split in the phylogeny, we do not expect to find a signal, while we have the most power to detect coevolution among genes that are frequently lost or gained via horizontal gene transfer and widely distributed among clades.

Genes that are frequently gained and lost can purely by chance generate a nonzero score. To test for this, we found the total number of discordances between close pairs for each gene. We then parameterized a Poisson Binomial distribution for each gene pair as follows: each close pair is modeled as an independent draw from a Bernoulli distribution. This draw represents the event of a “double discordance,” where the close pair is discordant for both genes in the gene pair. Under the null assumption of independence between the two genes, the probability of this event is the product of the probability of the event of a single discordance in each gene. We assume that the probability of a single discordance for each gene is proportional to the relative core genome distance between the two samples in the close pair, normalized across the total pairwise core genome distance across all pairs. The proportionality constant is the total number of discordances we observe across all close pairs for that gene. Thus, the expected number of discordances for each gene is equal to the observed number of discordances, and close pairs with larger pairwise distances are more likely to contain double discordances. We used a Bonferroni correction with $\alpha = 0.05$ on the resulting p-values from this Poisson Binomial distribution to determine statistical significance.

**Fig. 2:**
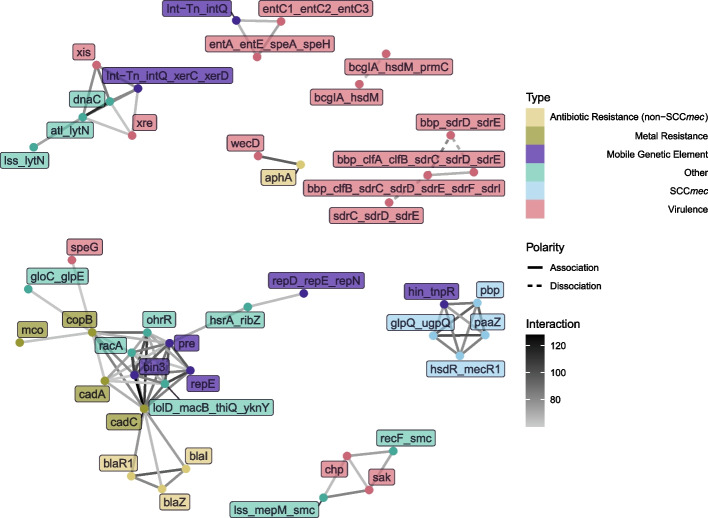
Gene-gene coevolution network for the top 77 significant gene pairs in the full dataset, with nodes colored by gene function, edge color indicating the strength of the inferred interaction, and edge type indicating the polarity of the interaction. A small handful of kinds of genes that are all frequently horizontally transferred—primarily relating to resistance, virulence, or gene transfer itself—tend to dominate the interaction network. The cluster involving *bbp*, *clf*, and *sdr* genes likely is a result of a limitation of automated annotation software, which appears in this case to have given different names to different versions of the same gene families. Thus, these interactions are negative because they represent alternate annotations

To construct interaction networks such as Figs. 2 and 4 , we chose a coevolution score threshold; if two genes have a score above this threshold, we drew a link between them with the weight being the score. These score thresholds were chosen primarily for visualization purposes, but they were always chosen from the extreme high end of the score distribution.

## Results

### Gene-gene interactions range from individual operons to complex webs

We consistently find some of the strongest signals of coevolution among genes related to resistance to antibiotics and metals; mobile genetic elements; and genes that influence virulence and toxicity, by e.g. producing a toxin, being involved in biofilm formation, or regulation. But the coevolution networks also include many genes whose functions do not obviously pertain to any of the aforementioned functions. Figure 2 provides an example of such an interaction network obtained from a full-dataset analysis, using only the top 77 significant scores that did not include unannotated genes.

There are eight total clusters of interactions in Fig. 2. The largest contains a non-SCC*mec* operon that confers beta-lactam resistance (*blaZ*, *blaI*, and *blaR1*), genes involved in resistance to the presence of cadmium (*cadA*, *cadC*)—reflecting a known plasmid interaction [40]—and copper (*copB* and *mco*), and genes involved in plasmid replication (*repD*, *repE*, *repN*, and *pre*). Another cluster primarily contains genes involved in the SCC*mec* cassette (*pbp* (*mecA*), *mecR1*, *paaZ* (*maoC*), and *upgQ*). Finally, there are six smaller clusters that contain a wide variety of virulence-related genes, including one pair involving an aminoglycoside resistance gene (*aphA*). These interactions paint a picture of recent genetic coevolution in *S. aureus* that focuses on host-pathogen interaction in all of its many facets.

### Most strong interactions are among physically linked genes, but many strong interactions are not

**Fig. 3:**
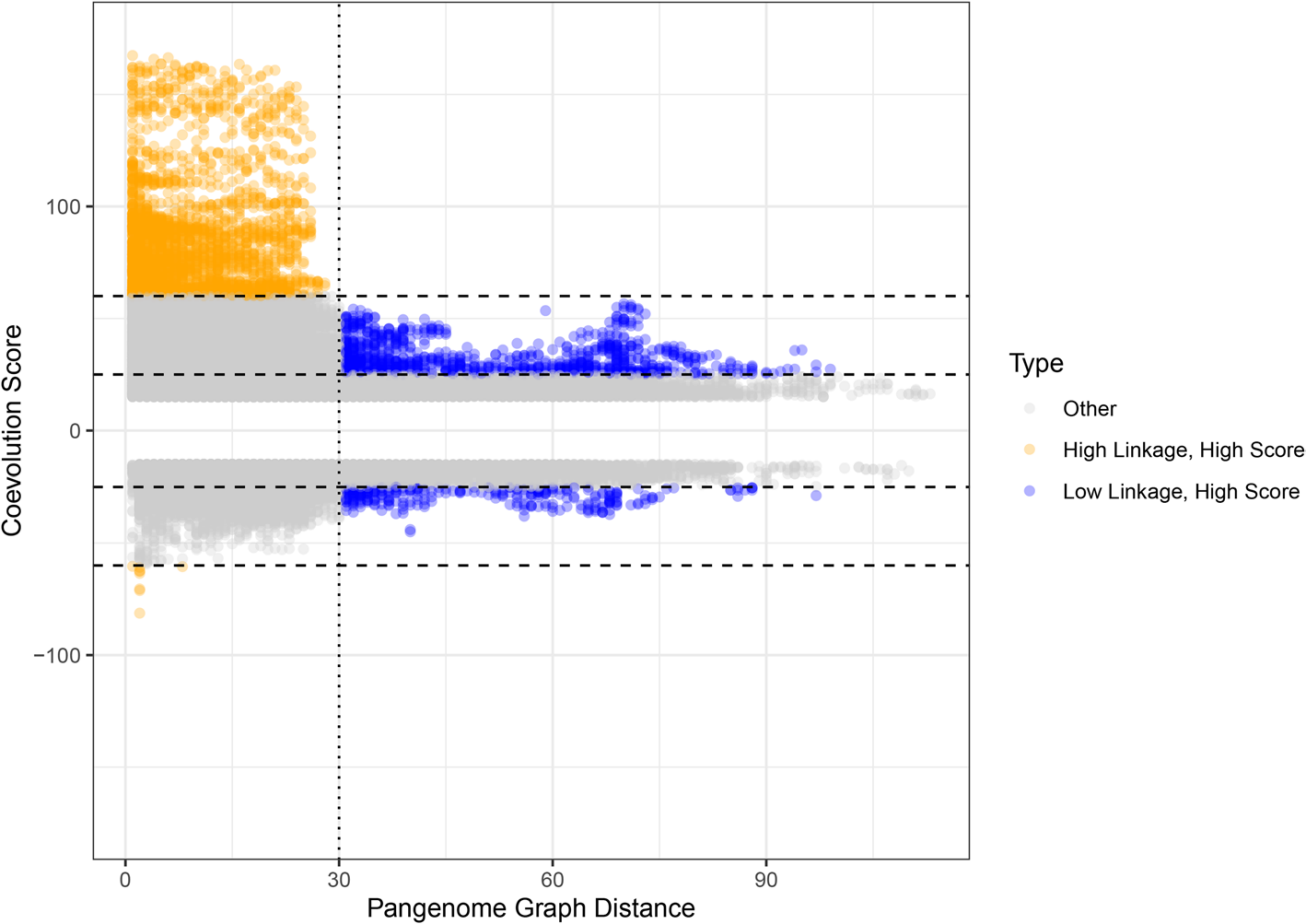
The strongest interactions are positive and occur between genes that are physically close together. However, strong interactions still occur between genes that are far apart. The horizontal axis is the shortest-path graph distance between a pair of genes in a pangenome graph, and the vertical axis is the coevolution score between those genes. The blue points indicate pairs that have score > 25 and a graph distance > 30 (the vertical dotted line), and the orange points indicate pairs that have score > 60 and graph distance < 30. All points with scores less than 15 were removed

Despite frequent gain and loss among the accessory genomes of *S. aureus*, we can compute the effect of physical linkage between genes using a pangenome graph from Panaroo [39]. This and other pangenome graph methods produce a network where each node is a homologous gene cluster and each edge represents at least one genome in which an element of the two gene clusters is adjacent on a contig. For our subsample of size 10,000, we computed the pangenome graph using Panaroo, and then used the shortest-path graph distance to quantify the amount of physical linkage. Figure 3 plots our coevolution score against this graph distance. Additional file 1: Fig. S7 (Section G) plots the overall distribution of scores, without pangenome graph distance.

There are four notable features of Fig. 3. First, the highest scores are restricted to graph distances that are under 30. Second, beyond graph distance 30, there appears to be little effect of graph distance on the maximum score. Third, there are still reasonably high scores beyond graph distance 30. Finally, fourth, there appears to be little effect of graph distance on negative interactions. The vast majority of interactions in Fig. 2 are in the high-linkage, orange regime in Fig. 3.

**Fig. 4:**
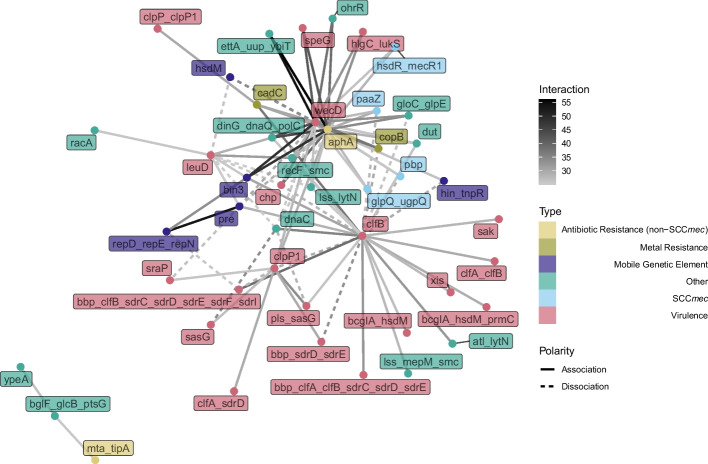
Gene-gene coevolution network for the top 94 significant gene pairs in the full dataset with graph distance > 30 and no unannotated genes, with nodes colored by gene function, edge color indicating the strength of the inferred interaction, and edge type indicating the polarity of the interaction. The interactions in this network are dominated by “spokes” around three genes–*aphA*, *wecD*, and *clfB*. In addition, there are many more negative interactions in this network than in Fig. 2

A network of low-linkage, high score interactions is presented in Fig. 4. In this case, low-linkage is defined as genes that are separated by a minimum of 30 genes in every sample genome. Thus, these genes are not found close together in the genome. Unlike in Fig. 2 (which represents the strongest overall interactions and almost every interaction is between genes that are separated by less than 30 genes in the pangenome graph) a substantial fraction of the interactions in Fig. 4—between genes that are not found close together— are dissociative. The structure of the graph in Fig. 4 is of a “hub-and-spoke” network, where most genes are connected by “spokes” to only a few central “hub” genes. Interestingly, two of the three genes that form the main “hubs” of this hub-and-spoke network (*aphA* and *wecD*) have a pangenome graph distance of 1, which means that they occur in at least one sample adjacent to each other. By definition, the interactions present in the network in Fig. 4 must occur when *aphA* and *wecD* are not adjacent. Thus, *aphA* and *wecD* must associate with a wide variety of genes, sometimes including each other and sometimes not. While *aphA* and *wecD* interact with genes of many types, the clumping factor *clfB* forms a hub that primarily involves other virulence genes, suggesting that *clfB* warrants further study in its relationship to antibiotic resistance and other forms of virulence.

**Fig. 5:**
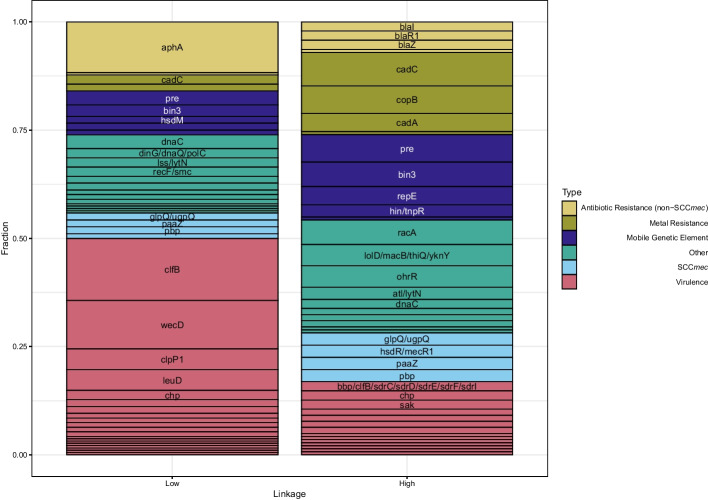
Genes found in highly-scoring interactions between linked genes differ in function from those found in highly-scoring interactions between unlinked genes. In high-linkage interactions, antibiotic resistance is more specific and varied, and SCC*mec*, mobile genetic element, and metal resistance interactions are more prevalent. In low-linkage situations, genes that are involved in virulence and pathogenicity are much more prevalent. The high-linkage scenario consists of all interactions that are orange (score > 60) in Fig. 3. The low-linkage scenario consists of all interactions that are blue (score > 25 and graph distance > 30) in Fig. 3. Homologous clusters of hypothetical genes were not included in this figure

Figure 5 describes the distribution of types of genes found in the orange (high-linkage, high-score) and blue (low-linkage, high-score) regions of Fig. 3. The high-linkage, high-score interactions involve more SCC*mec*, metal resistance, and mobile genetic element genes, as well as diverse array of antibiotic resistance genes. By contrast, the low-linkage, high-score interactions involve mostly genes related to virulence and pathogenicity. Thus, we see a pattern where virulence evolution is much more pleiotropic and able to reach beyond linkage restrictions. In contrast, antibiotic resistance is more restricted to being carried around in groups by mobile genetic elements.

The fact that we see less effect of linkage on the negative scores in Fig. 3 suggests a possible reason for the bias towards positive scores we detect: there is a specific mechanism for genes to be co-inherited or co-lost (i.e. linkage) which does not exist for genes to be inherited alternately.

### Antibiotic resistance phenotypes fall into two sets of interactions

**Fig. 6:**
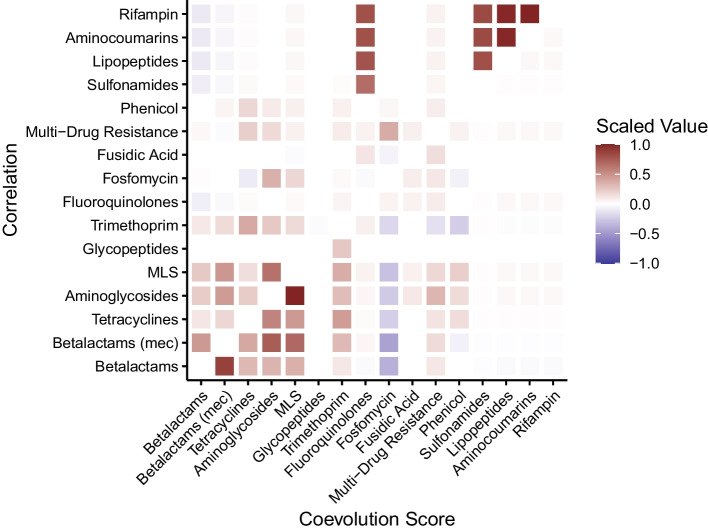
Significant coevolution scores (bottom-right triangle) and pairwise correlation values (top-left triangle) for predicted antibiotic resistance phenotypes in Staphopia. The strongest interaction block involves resistance to MLS, aminoglycosides, betalactams, and tetracyclines. Multi-drug resistance, and resistance to fusidic acid, glycopeptides, trimethoprim, fluoroquinolones, rifampin, lipopeptides, and sulfonamides have peripheral interactions. The axes are ordered according to a hierarchical clustering on the coevolution score

Our coevolution score is not restricted to gene presence/absence and can be applied to any binary trait. We initially applied the score to SNPs, but found that accessory genes had more interesting evolutionary patterns in this dataset. We can also apply our method to binary phenotypes, such as the presence or absence of antibiotic resistance. Staphopia predicts antibiotic resistance phenotypes using ARIBA [36]. For each sample in the full-dataset analysis, we computed coevolution scores for these predicted antibiotic resistance phenotypes. Because we did not need to annotate genes for this analysis, we were able to use the entirety of the 32,641 non-redundant samples in the dataset. Figure 6 displays a heatmap of the significant interactions and significant pairwise correlations for these phenotypes. Note that the coevolution scores are scaled so that the highest magnitude is one and the lowest magnitude is zero.

There is a strong positive interaction cluster between both beta-lactam resistance phenotypes, MLS, aminoglycoside, trimethoprim, tetracycline, and phenicol resistance. The two strongest interactions are between aminoglycoside and MLS resistance and between SCC*mec* and non-SCC*mec* beta-lactam resistance. Fosfomycin resistance appears to strongly negatively interact with the other resistances. Finally, the remaining resistance phenotypes form a peripheral, weakly interacting group. These phenotypes are also in general much rarer than those in the beta-lactam interaction group, so their signal is limited.

The high-scoring group also has high correlation, but fosfomycin resistance has a clear negative signal with the coevolution score and no clear signal with correlation. Five of the peripheral resistance phenotypes are strongly correlated with each other, but have very little signal with the coevolution score.

### Comparison with other methods

In this section, we compare DeCoTUR to two other existing methods—SpydrPick [34] and Coinfinder [31]—on datasets of 100, 500, 1000, and 5000 samples. The 5000 sample dataset was randomly subsampled from the 10000 samples used for the full dataset analysis. The subsequent samples are nested subsets of the 5000-sample dataset. We also fixed the number of genes in each sample by selecting intermediate-frequency genes symmetrically by rank around the median frequency gene to get 1000, 3000, 5000, and 10000 genes for each sample size. The Panaroo implementation of SpydrPick can be run with or without the ARACNE analysis that is the signature of SpydrPick. We show results from both options. To get full results, Coinfinder must be run twice: once for association and once for dissociation. Our runtimes add the two times up. There were a number of choices we made for using DeCoTUR in this comparison. First, we ran DeCoTUR for three different numbers of close pairs: 100, 500, and 1000. We also only computed significance for the top 10% of scores. Finally, we used block sizes of 100 for the score computation and 1000 for the significance tests. These choices were not optimized for any particular outcome.

**Fig. 7:**
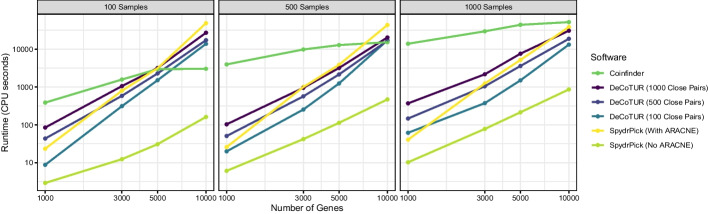
DeCoTUR is comparable in runtime to SpydrPick, and is faster than Coinfinder except when there are a small number of samples and a large number of genes

Figure 7 provides the runtime results for each of these methods on each of these datasets, when possible. The machine used in this comparison had 12 cores, an Intel Core i7-8700 CPU, and 64 GB of RAM and ran Ubuntu and Windows. Scripts for running each of these packages are provided in the supplementary material. DeCoTUR has the benefit of being relatively unaffected by sample size (by design), whereas Coinfinder has the benefit of accommodating large numbers of genes relatively efficiently. SpydrPick without ARACNE is the fastest method overall, and SpydrPick with ARACNE is comparable to DeCoTUR. Using different numbers of close pairs, it is possible for DeCoTUR to span the range of runtimes from SpydrPick with no ARACNE to Coinfinder. Additional file 1: Fig. S5 demonstrates that the coevolution score does not greatly depend on the number of close pairs used. Users can therefore choose the largest number of close pairs that accommodates their computational resources and their biological question.

**Fig. 8:**
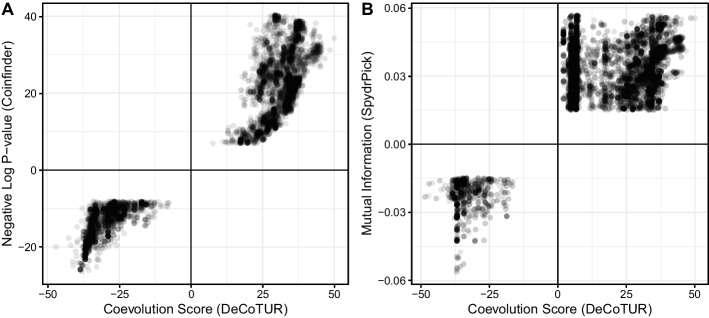
Comparison of the coevolution score found by DeCoTUR with (A) the negative log-p-value of the association found by Coinfinder and (B) the mutual information computed by SpydrPick (without the subsequent ARACNE step). Only coevolution scores that were significant were used. In both cases, there is a general positive correlation between the methods, though they each have different filtering cutoffs. The relationship is less strong with SpydrPick, which is perhaps because many spurious associations were not filtered out by ARACNE (though running the ARACNE step on a dataset of this size removed all the interactions). Note that Mutual Information is an always-positive quantity, so the values that corresponded to negative coevolution scores were artificially labeled as negative

Figure 8 shows how the scores found by SpydrPick (without ARACNE) and Coinfinder compare to those found by DeCoTUR for 500 samples, 1000 genes, and 500 close pairs. All three methods are generally positively correlated with each other. The relationship between SpydrPick and DeCoTUR is less strong (Pearson correlation 0.77) than that between Coinfinder and DeCoTUR (Pearson correlation 0.95) (we note that these relationships are not obviously linear so correlation coefficients are only presented here to quantify the clearly-seen visual differences between the two.). This difference is likely due to the fact that non-spurious results were not removed from the SpydrPick results because the ARACNE step removed all interactions, and so we did not include that step in this analysis.

## Discussion

We have presented a new method for detecting interactions between genes in large bacterial datasets, using pairwise divergence in the core genome to find closely-related pairs of organisms and finding pairs of genes that differ within the same close pairs. We applied this method to Staphopia, a dataset of more than 40,000 genomes of *Staphylococcus aureus*, to find a network of accessory genes that are being gained and lost together.

The gene interactions that our method detects present an interconnected picture of various ways in which *S. aureus* interacts with its environment. Along with antibiotic resistance genes, we found substantial interaction with genes that promote virulence and pathogenicity—ranging from host colonization to toxin production—as well as genes that code for resistance to metals and genes that are involved in plasmid replication, bacteriocins, and DNA metabolism. Our results suggest that recent gene-gene coevolution in *S. aureus* is a complex, interconnected web in which horizontal gene transfer allows lineages to rapidly acquire a suite of traits involved in pathogenicity, including antibiotic resistance, host colonization, and competition with other bacteria.

We found that most interactions between pairs of genes are positive, with the presence of one gene correlated with the presence of the other, rather than anti-correlated. This is similar to the result found by [41] using a different method (Coinfinder) in a different system (*E. coli*), suggesting that it may be a general pattern. Both of these results support the idea that HGT-based evolution is driven more by the collection of genes that work well together as opposed to the sorting of a diverse set of genes that are interchangeable. Of course, selection may favor linking such sets of genes into operons, which will then facilitate their co-transfer and strengthen the pattern of positive associations.

Using distance in a pangenome graph as a proxy for physical linkage, we found that the strongest interactions were found with the most strongly-linked genes (i.e. pangenome graph distance less than 30). These strong interactions were almost all positive, suggesting that the positive bias in the strongest scores is indeed driven by the transfer of operons of genes. Negative interactions were much more prevalent as a fraction of total strong interactions for pairs of genes that were further than 30 genes apart in the pangenome graph, suggesting that these interactions are driven more by pleiotropy than by co-transfer events. High-linkage interactions were enriched in metal resistance, mobile genetic element, and slightly in antibiotic resistance genes than low-linkage interactions. By contrast, low-linkage interactions were enriched in virulence-related genes. These patterns suggest that interactions between resistance genes are driven by direct HGT, whereas interactions between virulence genes are driven by epistasis and pleiotropy.

One of the more unexpected results we found was cadmium resistance’s frequent strong coevolution with antibiotic resistance. It is not obvious why these genes should have such a strong signal across clonal complexes, especially considering that there are other genes that are also frequently found in SCC*mec* that show much weaker interaction. One potential explanation could involve a linkage of cadmium resistance to survival in wastewater as a transmission mechanism [42].

Interactions between antibiotic resistance phenotypes can be divided into two groups: a major group with common resistance phenotypes centered around beta-lactam resistance, and a minor group with rare resistance phenotypes. Although our overall results suggest that antibiotic resistance is transmitted via operons in general, some phenotypes show unexpected patterns (such as fosfomycin, which has a negative coevolution score with the other resistance phenotypes in the major group).

The Staphopia database is compiled from public data; sampling biases in these data will therefore be preserved in Staphopia. One major such bias is the overabundance of MRSA (methicillin-resistant) vs. MSSA (methicillin-sensitive) strains due to the important clinical relevance of certain MRSA strains. This bias could potentially inflate the importance of the SCC*mec* cassette. By downweighting bushes by their size, we avoid the score being dominated by a recent well-sampled branch of the tree. An additional possible solution to this bias would be to downsample to reduce MRSA frequency within the sample. Ultimately, more sampling of MSSA strains is necessary to properly understand *S. aureus* evolution. In addition, there is a tradeoff inherent in the Staphopia database where shotgun-assembled genomes are used, resulting in a decrease in the quality of automated gene calling, annotation, and linkage estimate. Thus, even though we use automated annotation via Panaroo, there are still limitations to this method. In the future, large amounts of complete genomes—such as from Oxford nanopore or PacBio sequencing—may improve the picture.

The ability to discover and investigate interactions between genes in bacteria will only increase with the increase in the accessibility of large amounts of data provided by databases such as Staphopia. With more data, we may be able to discover more interactions with smaller signals, or interactions that are strong but rare. We constructed our method specifically to be able to keep up with this progress. Methods such as ours, coupled with databases such as Staphopia, will allow both the study of broad-scale patterns of bacterial evolution as well as providing more focused results for future study.

## Supplementary Information

**Additional file 1**. Supplementary results and figures.

## Data Availability

All code and data can be found at https://github.com/rohansmehta/DeCoTUR_manuscript_code.
