# Supplementary material for: Detecting patterns of accessory genome coevolution in Staphylococcus aureus using data from thousands of genomes
Source: BMC Bioinformatics. 2023 Jun 9;24:243. doi: 10.1186/s12859-023-05363-4 (PMC10251594; doi:10.1186/s12859-023-05363-4)
Supplement: Supplementary file 1 — Additional file 1. Supplementary results and figures. [file 12859_2023_5363_MOESM1_ESM.pdf]

A Sample sizes for each clonal complex.

Figure S1 displays the sample sizes for each clonal complex in this dataset, both prior to and after removal of samples that were identical across both the core genome and in accessory gene presence/absence.

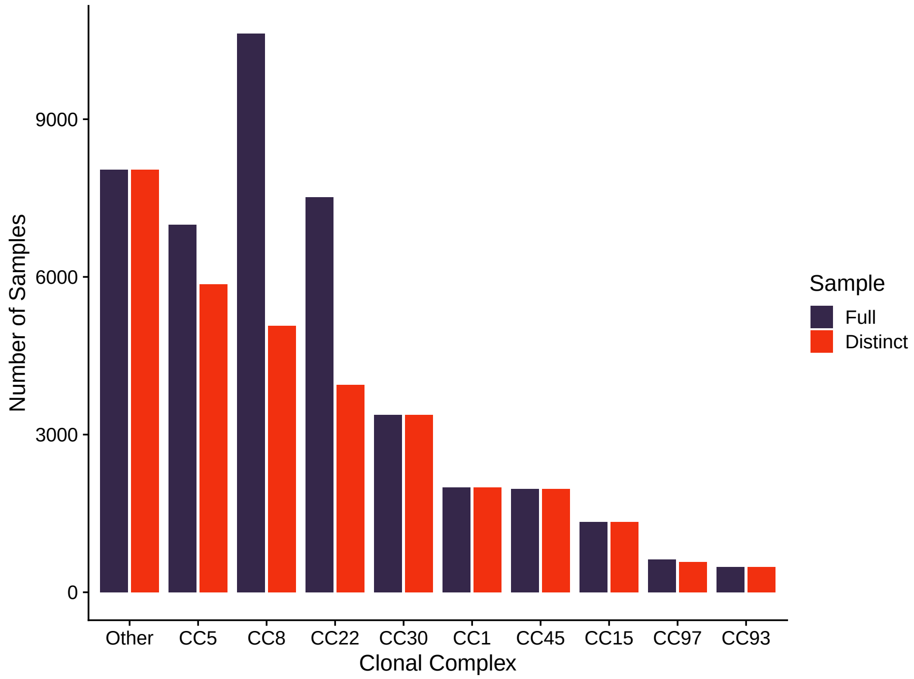

Figure S1: The total number of public samples per clonal complex in Staphopia (“Full”), as well as the total number of samples obtained after removing samples that were identical across both the core genome and accessory gene presence/absence (“Distinct”).

B Distance scales and number of close pairs for each clonal complex

For our data, the individual clonal complexes varied as to the scale of divergence they comprised. Figure S2 displays the pairwise distance distributions for all distinct samples in each clonal complex, along with example distances cutoffs that would lead to 5000 close pairs chosen from each clonal complex, for reference.

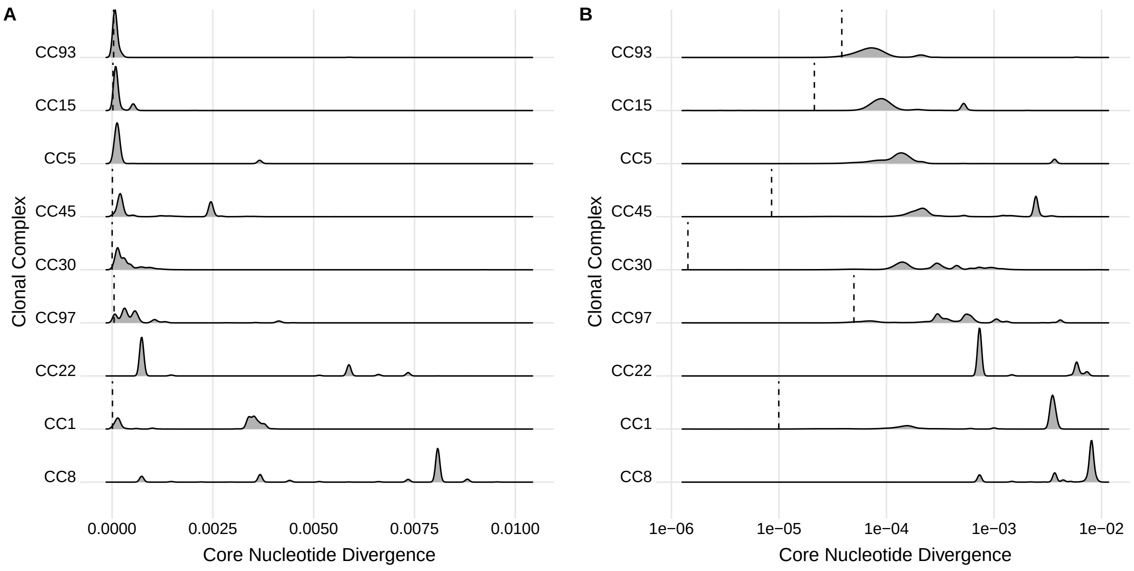

Figure S2: Pairwise nucleotide divergence in the core genome of Staphopia samples, where all zero values were excluded. (A) Linear divergence scale. (B) Log divergence scale. Distance cutoffs that would lead to 5000 close pairs from each clonal complex are shown in the vertical dashed lines. CCs 5, 8, and 22 have sufficiently many zero values to make the distance cutoff zero, therefore not shown.

C Choosing close pairs

Because scales of divergence vary dramatically between clonal complexes (Figure S2), simply choosing a single distance cutoff using all nonredundant samples from the dataset will bias the set of chosen samples towards dramatic overrepresentation of some clonal complexes but not others. Choosing a larger distance cutoff leads to a more representative sample (Figure S3).

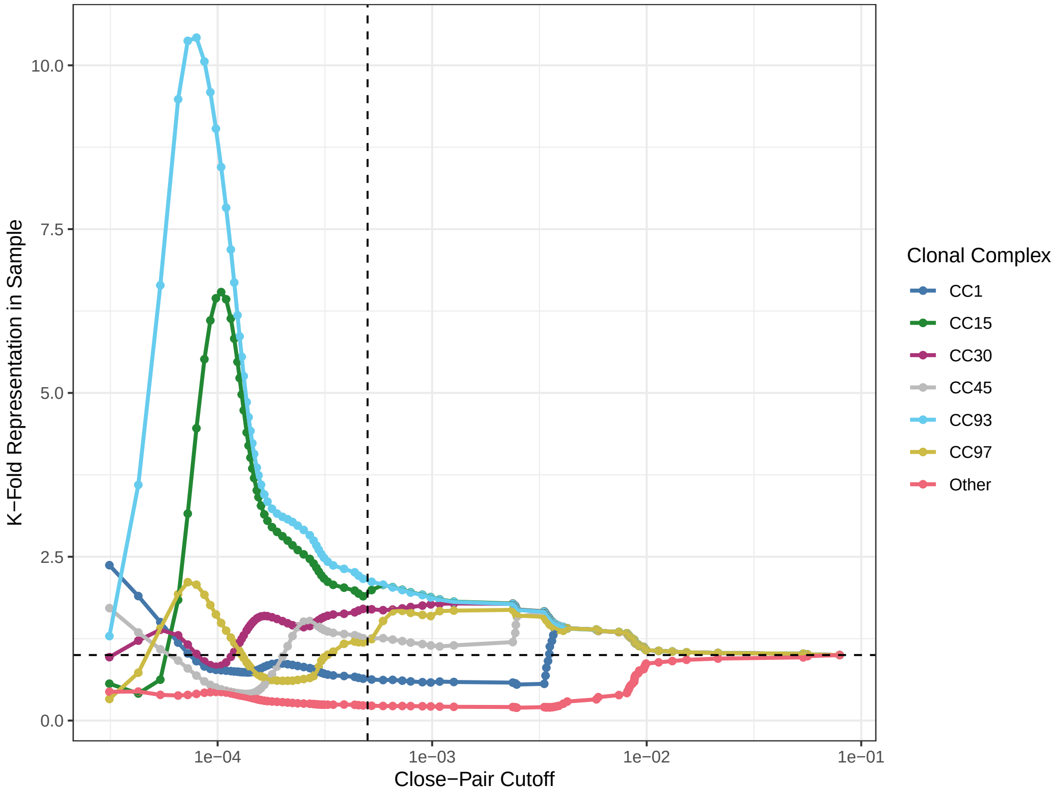

Figure S3: Choosing a core-genome distance cutoff across the whole Staphopia dataset overrepresents some clonal complexes and underrepresents others. The y-axis represents the ratio of the fraction of samples in each clonal complex below the corresponding distance cutoff to the fraction of samples in each clonal complex in the whole dataset. Excluded from this set of clonal complexes are CC5, CC8, and CC22, which have substantial numbers of samples that are identical in the core genome. The vertical line represents the distance cutoff chosen for the full dataset analysis.

On the other hand, choosing a larger distance threshold results in an enormous number of close pairs that render the procedure to compute coevolution scores computationally intensive. To combat this problem, we chose a compromise distance threshold of 0.0005 (vertical line in Figure S3). In choosing this distance threshold, we did not consider CC5, CC8, or CC22; these clonal complexes had many samples that were identical in the core sequence. For the other clonal complexes, we found that there were approximately 900,185 close pairs at this distance cutoff, resulting in 128,598 close pairs per clonal complex on average. For CC5, CC8, and CC22, we randomly sampled 128,598 close pairs each from the set of close pairs that were below the distance cutoff, resulting in a total of 1,285,976 close pairs. From this total set of close pairs, 187,311 involved samples we included in our subsample of 10,000 for the full dataset analysis. Of these, we randomly sampled 10,000 to serve as our set of close pairs for the full dataset analysis.

D Number of discordant close pairs per gene

Each gene can differ in presence/absence state for any close pair of samples. These discordances are tallied and turned into the coevolution score. Figure S4 displays the distribution of discordances for the genes in our full dataset analysis over 10,000 total close pairs of samples. All named genes with over 2000 discordances are: *atl/lytN, lss/lytN, dnaC, clpP1, bbp/clfA/clfB/sdrC/sdrD/sdrE, clfA/sdrD, ermC, immR/lexA, xis, pre, recF/smc*, and *clfB*.

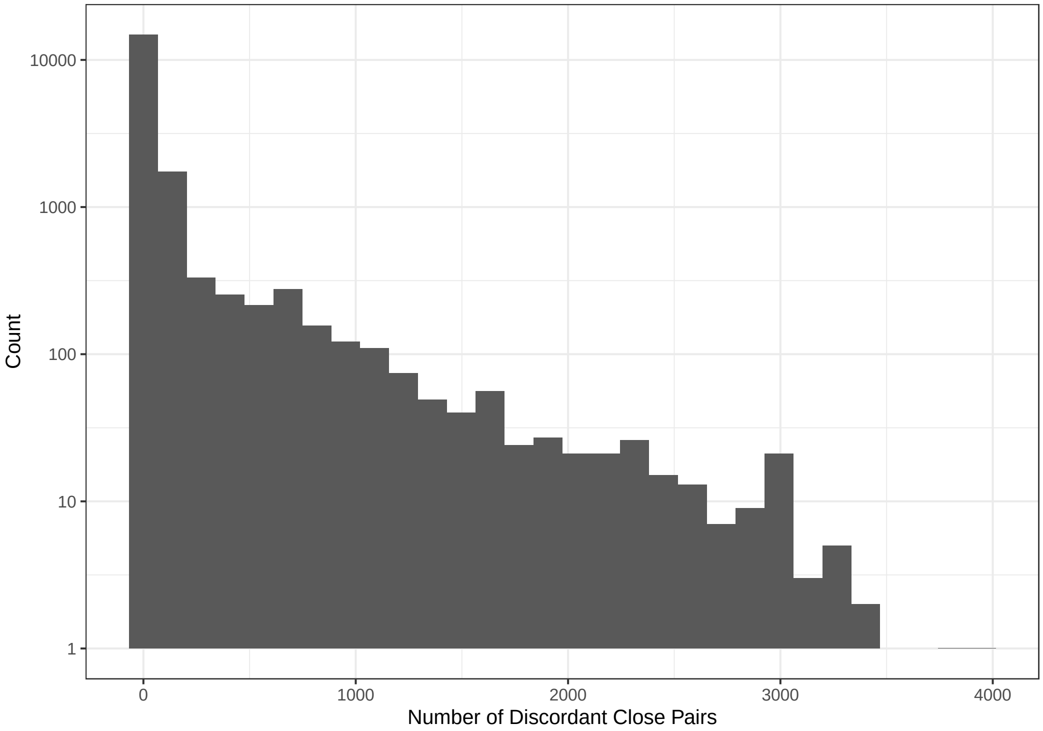

Figure S4: The number of close pairs for which each gene is discordant. The linear nature of this distribution on a log y-axis indicates that these discordances are exponentially distributed, with the maximum number of discordances about 3500, or 35% of the 10,000 close pairs.

E Coevolution score does not systematically depend on the choice of distance cutoff

To study how the choice of number of close pairs affected the coevolution score, we computed the coevolution score for an example dataset that used a random sample of 500 samples and 1000 genes from the full dataset over three choices of close pair number: 100, 500, and 1000. This was one of the datasets used in the section above comparing DeCoTUR to other methods. Figure S5 demonstrates that the number of close pairs chosen does not dramatically effect the coevolution score. In fact, no gene pairs with significant scores changed polarity across this numerical experiment.

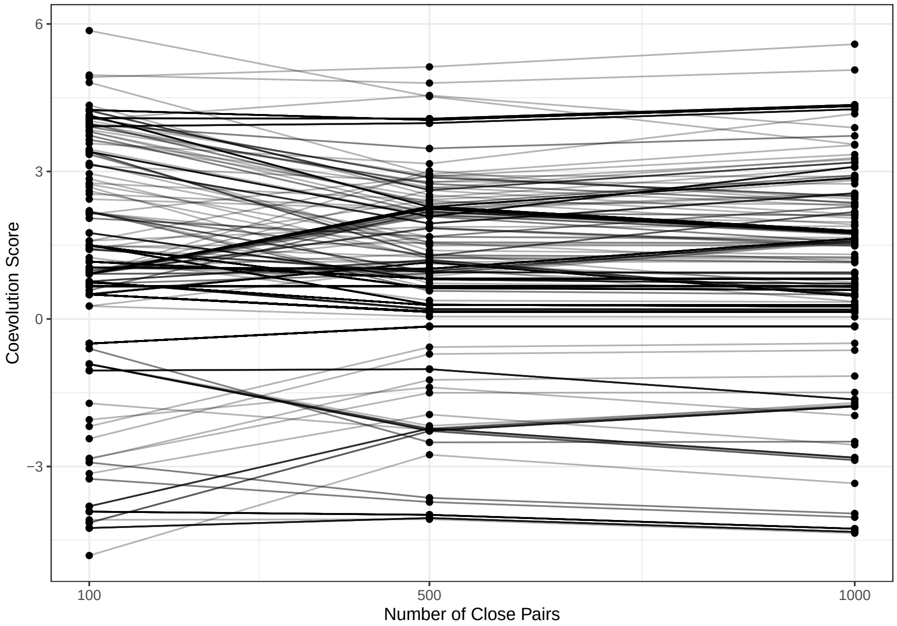

Figure S5: The choice of the number of close pairs does not systematically affect the coevolution score, and the highest scores remain high across a wide range of cutoffs. Shown here is a random subsample of 10,000 gene pairs from a sample of 500 species and 1000 genes. Gene pairs were sampled from the set of all gene pairs where scores across all three choices for the number of close pairs were significant.

F Example phylogenetic tree for CC1

Figure S6 displays the phylogenetic tree for CC1, the clonal complex with the largest sample size in Staphopia. The bushy structure is seen, with the vast majority of samples belonging to a handful of very-recently-diverged bushes.

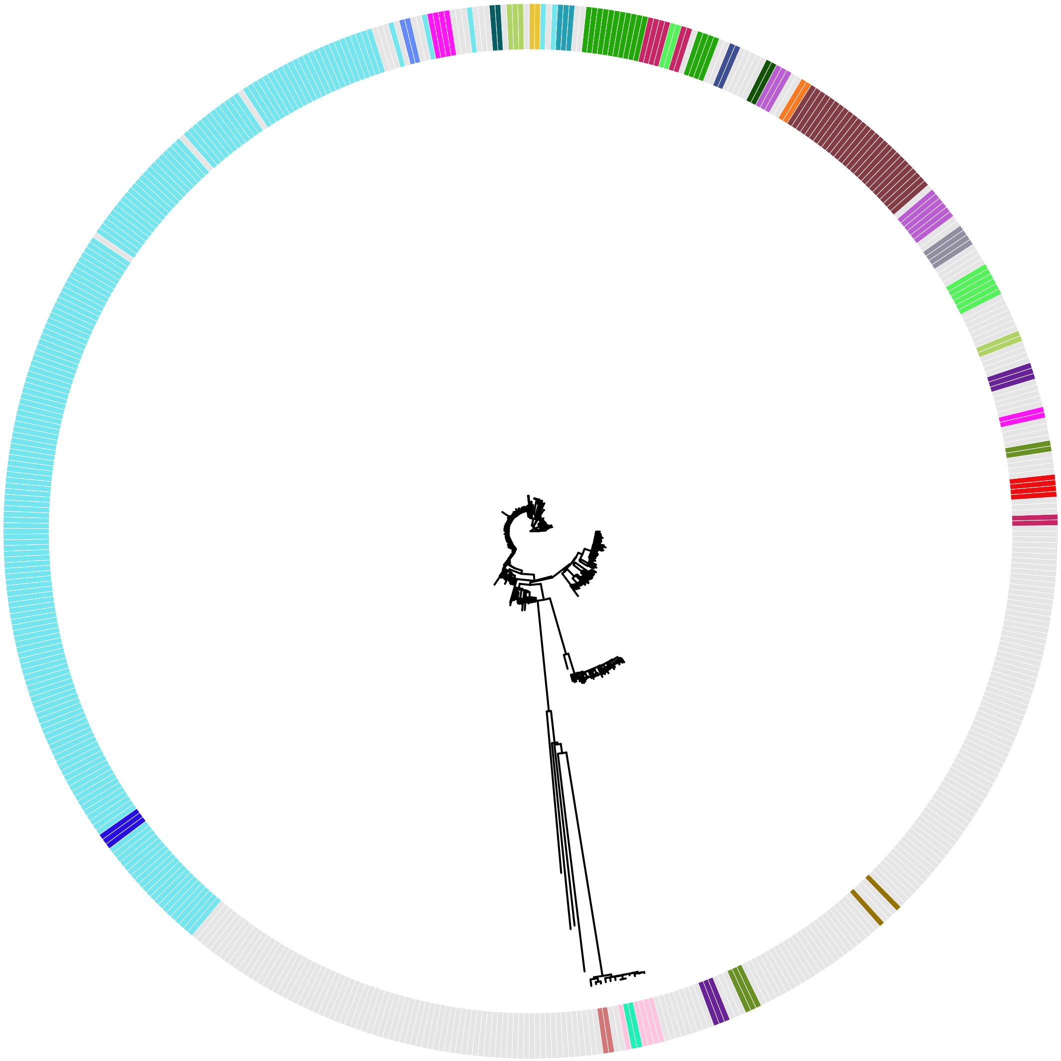

Figure S6: The phylogenetic tree of CC1 consists of bushes of very closely-related samples separated by long branches. This bushy structure makes the specific relationships between samples within a bush subject to resolution error. Colors distinguish separate bushes for close pairs at distance cutoff 5×10^{-5}. Samples not included in a close pair are not colored and are in gray.

G Distribution of scores

Figure S7 displays the overall distribution of scores found in Figure 3. Negative associations are depicted with negative numbers here. The cutoffs used in Figure 3 (±60 and ±25 are shown in dashed lines in this figure. Note the clear bias towards positive scores. The exponential drop-off away from 0 is slower for positive scores, and flattens out for the highest positive scores, indicating both an overall bias towards positive association and an overrepresentation of positive scores in the extreme parts of the distribution.

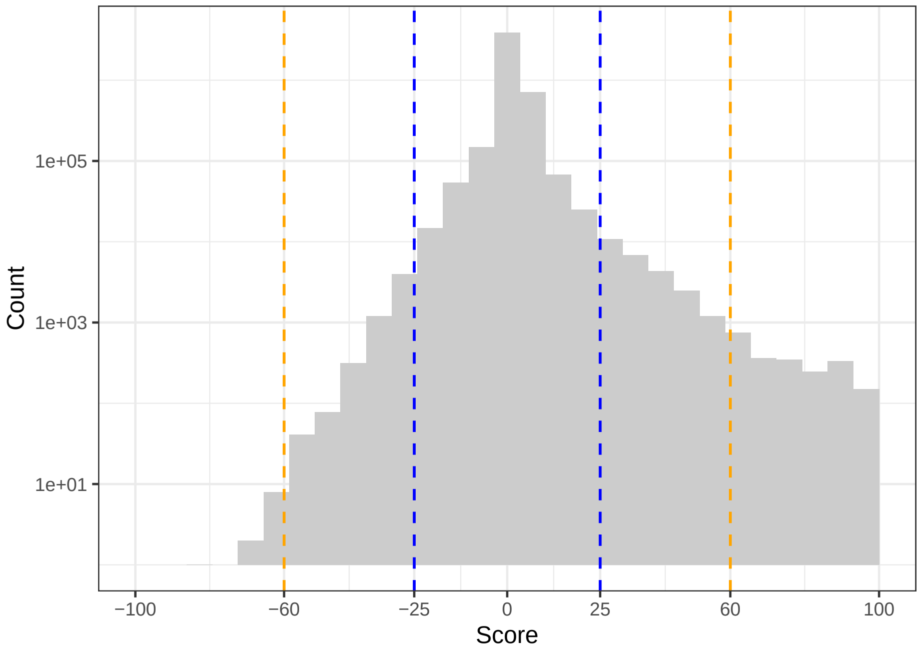

Figure S7: The overall distribution of scores in Figure 3 is concentrated around 0, with exponential dropoff on both sides. Note that the y-axis is on a log scale. The cutoffs in Figure 3 are shown here with dashed lines colored by the colors of the corresponding regions they delineate in Figure 3. in Figure 3. There is a clear bias towards positive association overall.
